# Privacy-Preserving Synthetic Data Generation Method for IoT-Sensor Network IDS Using CTGAN

**DOI:** 10.3390/s24227389

**Published:** 2024-11-20

**Authors:** Saleh Alabdulwahab, Young-Tak Kim, Yunsik Son

**Affiliations:** 1 Department of Computer Science and Engineering, Dongguk University, Seoul 04620, Republic of Korea; saleh@student.dongguk.edu; 2 Department of Radiology, Massachusetts General Hospital, Harvard Medical School, Boston, MA 02114, USA; ytkim@mgh.harvard.edu; 3 Department of Biomedical Sciences, Korea University College of Medicine, Seoul 02841, Republic of Korea; 4 Division of AI Software Convergence, Dongguk University, Seoul 04620, Republic of Korea

**Keywords:** differential privacy, data utility, generative adversarial network, intrusion detection systems, Internet of things, deep learning

## Abstract

The increased usage of IoT networks brings about new privacy risks, especially when intrusion detection systems (IDSs) rely on large datasets for machine learning (ML) tasks and depend on third parties for storing and training the ML-based IDS. This study proposes a privacy-preserving synthetic data generation method using a conditional tabular generative adversarial network (CTGAN) aimed at maintaining the utility of IoT sensor network data for IDS while safeguarding privacy. We integrate differential privacy (DP) with CTGAN by employing controlled noise injection to mitigate privacy risks. The technique involves dynamic distribution adjustment and quantile matching to balance the utility–privacy tradeoff. The results indicate a significant improvement in data utility compared to the standard DP method, achieving a KS test score of 0.80 while minimizing privacy risks such as singling out, linkability, and inference attacks. This approach ensures that synthetic datasets can support intrusion detection without exposing sensitive information.

## 1. Introduction

**Figure 1:**
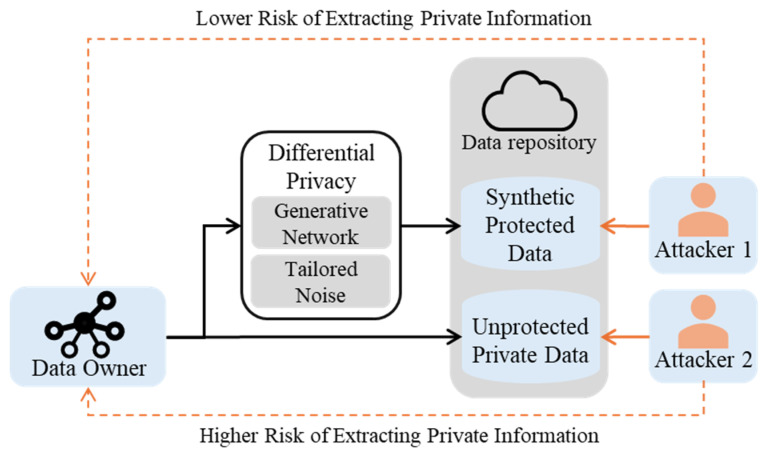
The threat model of privacy attacks.

Big data plays a significant role in machine learning (ML) and deep learning (DL). IoT networks generate valuable information that can be used to train ML-based intrusion detection systems (IDSs) [1]. However, collecting and distributing these data might lead to privacy risks, especially if the data are transferred to the cloud or the creation of ML-based IDSs is outsourced. Thus, institutions need methods like differential privacy (DP) to anonymize datasets and prevent re-identification without compromising their usability [2]. Figure 1 presents the threat model when the data owner wants to pass the dataset for the downstream task or to a data repository where a wider number of users can access it. Suppose the private data have not been protected by a DP method, as with attacker 2. In that case, it might face a privacy risk, like extracting private information using attacks such as attribute inference. Attackers can exploit seemingly unrelated attributes to infer private information indirectly. These attributes may not appear directly connected to sensitive data, but they can be revealed through advanced analysis. Performing the DP method aims to reduce the risk of these privacy attacks.

In ML and DL research, dataset quality is crucial for the performance of DL models. Selecting diverse, high-quality datasets is essential for training effective DL models [3]. Collecting datasets from real sources provides realistic results that can represent real-life applications. However, this only applies to ideal scenarios; real datasets can be unbalanced, small due to limited resources, and hold private information. Therefore, researchers have proposed several methods to solve these issues, such as augmentation, oversampling, and deep generative models.

Deep generative models use neural networks to learn the characteristics of a dataset and can generate synthetic data that closely resemble real data. One technique is using generative adversarial networks (GANs), which are a type of model that uses two neural networks: the generator, where new data are created, and the discriminator, which determines whether the data generated is real or fake. While GANs can generate high-quality datasets, further development is needed to match the requirements of target datasets and DL tasks [4].

ML-based IDSs greatly benefit from GAN models. They can generate more unseen attack scenarios, enabling the IDS model to learn from more extensive possibilities. Moreover, GAN models like conditional tabular GAN (CTGAN) effectively learn the attacks’ complex data distributions and hidden features. This can be beneficial as some attacks occur less often than others; hence, they can create more instances of these rare attacks with high quality and resemblance [5]. Creating ML-based IDSs requires a collective effort from the data provider, data analysts, and artificial intelligence experts. However, this requirement introduces privacy challenges as the collected data from the internal network can reveal sensitive information about entities. Therefore, data sharing needs to be carried out under a controlled framework [6].

Several challenges related to applying DP, generating synthetic datasets using DL methods, and safe sharing in the IoT environment. IoT devices have limited storage and computational power, especially in the context of energy constraints and a cost-effective ecosystem. Due to this, they cannot store and process the collected data; they usually transfer it to more powerful devices, such as third-party cloud service providers [7]. However, outsourcing raises significant privacy concerns, as unauthorized entities can compromise the privacy of an organization that owns the IoT environment and reveal confidential information. Hence, privacy preservation actions such as DP are essential before transferring private data outside the local network.

In differential privacy, the tradeoff between utility and privacy often reduces the similarity between the synthetic and original datasets, but increasing this similarity too much can introduce privacy risks [8]. Overprocessing noisy data to match the original dataset’s structure better may lead to overfitting, inadvertently revealing sensitive information [9]. This is why preserving key aspects of data distribution and structures such as correlations, variances, ranges, and outliers becomes essential. By carefully maintaining these characteristics, we can enhance the utility of the data without compromising privacy or falling into the trap of overfitting. To address these challenges, the novel contribution of this study is as follows:

- The integration of a utility-preserving DP with CTGAN to generate data using controlled noise injection instead of direct or random noise injection while maintaining data utility. When applying DP, smooth sensitivity and dynamic adjustment mechanisms are utilized to prevent excessive utility damage and affect the quality of the generated data by CTGAN.
- To maintain the distribution and structure, the method explores advanced utility–privacy balancing using quantile matching and dynamic KS adjustment to help preserve the original dataset’s statistical properties, structure, and distributions while avoiding privacy risks.
- The study considers the use of privacy risk metrics to test the efficiency of the DP methods. The proposed method outperforms the standard DP technique by achieving a more effective balance between utility and privacy. Unlike standard DP methods, which apply Laplace, exponential, and Piecewise noise levels across data features, our proposed DP shows a KS test score of 0.80, indicating high fitness between the synthetic and original data distributions. Privacy risks are effectively minimized, with singling out at 0.0069, linkability at 0.001, and inference at 0.35, ensuring robust privacy protection while preserving significant data utility.

**Figure 2:**
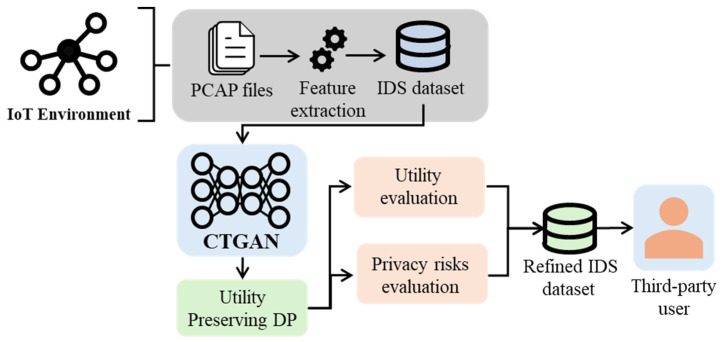
An overview of the proposed utility-preserving DP methodology.

Figure 2 shows an overview of the proposed methodology. After generating the synthetic dataset, the utility-preserving DP (UP-DP) is applied to it. Then, the dataset’s utility and privacy risks are evaluated before the refined dataset is passed to the third-party user for further procedures and tasks such as data analysis and DL tasks.

The rest of the article consists of the following: Section 2 describes recent studies on integrating privacy-preserving techniques with synthetic data. Section 3 is the methodology, representing the proposed technique to achieve the study’s objectives. Then, Section 4 presents the proposed methodology’s utility and privacy measures. After that, Section 5 discusses the results, limitations, and potential implications. Section 6 is the conclusions; it summarizes the overall study and provides future directions.

## 2. Related Works

Some studies have used deep learning (DL) data generators as a privacy-preserving technique on their own without incorporating additional methods like differential privacy. Others have integrated these generators with different techniques, such as statistical models or other DL-based methods, to enhance privacy protection. This section will introduce different approaches to achieve the goal of generating high-quality and private datasets.

### 2.1. GAN Models for Privacy Preservation

As the studies [10,11] have suggested, using GAN models can be a promising tool for preserving privacy. It can be used to solve class imbalances and privacy challenges. Researchers proposed several novel types of GAN methods for tabular data, such as CouplaGAN [12], tableGAN [13], and tabular GAN (TGAN) [14]. These types of tabular data generators have been thoroughly studied in recent years in terms of their ability to generate datasets that represent real datasets and how private these generated datasets are. A study by Rajabi and Garibay [15] focused on the fairness aspect of the synthetic data. The goal of fairness in this study is to ensure that the synthetic data do not show a bias on protected attributes like race and gender, which can help in making decisions independently from these attributes. To achieve this, they proposed fair tabular data generation with GAN (TabFairGAN). This two-phase GAN model is trained to achieve data accuracy and then incorporates a fairness constraint to minimize discrimination based on protected attributes by modifying the loss function to reduce discrimination scores between protected groups. A study that tested the potential of GAN models in the privacy–utility tradeoff was conducted by Nik et al. [16]. They studied different state-of-the-art generators on medical datasets. The study also found that using a distance-based privacy metric, CTGAN was the best in privacy preservation.

A study by Sakib and Ghosh [17] compared a variation in GANs, CTGAN, and Wasserstein GAN (WGAN) with and without gradient penalty. Then, they compared them with a statistical method data generator, the Gaussian mixture model. They found that CTGAN showed the highest performance in most datasets regarding the privacy–utility tradeoff. These studies discovered the potential of using GAN-based generators to ensure the utility and privacy of the datasets. Their proposed methods showed promising results. However, advanced privacy-preserving methods need to be integrated with the CTGAN to enhance the model’s ability to provide synthetic datasets with higher privacy guarantees while maintaining utility. Moreover, the GAN methods need to be tested against privacy risks and attacks to ensure the balance of the synthetic datasets in the privacy–utility tradeoff.

### 2.2. Integrating Privacy Preservation with Data Generators

Researchers integrated differential privacy and federated learning into the generator’s framework to improve privacy preservation, according to a study by Fang et al. [18]. During the training, they added gradient clipping to stay within fixed bounds and noise injection sampled from a normal distribution. They concluded that their method can generate usable synthetic data and is robust against privacy risks. However, the privacy measures affected the performance of the CTGAN model. Venugopal et al. [10] proposed privacy-preserving GAN (pGAN); they applied batch normalization in the generator and dropout in the discriminator to improve convergence. Their method outperformed other GAN models, like TGAN, in the balance between utility and privacy. However, the data generated were not tested against privacy risks like inference and linkability attacks. Another study that integrated DP with CTGAN was conducted by Sun et al. [19]. They proposed a differentially private conditional GAN (DP-CGAN). They focused on preserving the variable dependencies and privacy in health datasets by injecting Gaussian noise into the gradients during training. They found that their proposed method was better than the other methods, such as CTGAN and TableGAN, in capturing the dependencies in the imbalanced datasets. Although the performance was promising, adding noise directly may lead to instability as Gaussian can obscure the pattern and dependencies in the data. A paper by Hindistan and Yetkin [20] focused on using CTGAN with DP on the sensitive features by adding Laplace distribution noise. However, random noise complicates the balance between privacy and utility, forcing a more tailored approach for better data quality.

Some studies used advanced statistical methods for data generation instead of DL methods like GAN. A study by Almeida et al. [21] proposed a model that combines Uniform manifold approximation and projection (UMAP) and an extension of the synthetic minority oversampling technique for nominal and continuous data (SMOTE-NC) to generate privacy-aware synthetic data. They first reduced the data into two directions using UMAP. Then, they mixed nearby points using the SMOTE technique to generate the synthetic data, ensuring privacy and diversity. Finally, they mapped the data to its original form for further analysis. They found that their generated dataset can be effectively used for machine learning tasks, outperforming the SMOTE-NC without UMAP. Yet, the generated dataset has not been tested by performing privacy attacks. Kroes et al. [22] proposed the use of a cluster-based synthetic data generation (CBSDG) method to generate a private and high-quality dataset. They performed this method in a blood transformation dataset. They first grouped similar blood donors into clusters and removed the personal details to protect privacy. After that, synthetic data were generated by sampling from these clusters. They found that although their method can produce datasets with high performance in terms of utility and privacy, some categorical attributes might still be inferred using privacy attacks. Hence, additional privacy measures and tests need to be taken.

### 2.3. Related Works Summary

Researchers have tested several DL and statistical methods to generate high-quality datasets. Some of them integrated DP methods to enhance the privacy preservation of the datasets. Although these studies showed promising results and contributed to this domain with innovative methodologies, further improvements are needed to solve the challenge of the utility–privacy tradeoff.

Table 1 summarizes the related studies presented. Some studies should have considered integrating advanced DP methods with the generators, and some of them directly added noise to the generator, which might lead to instability and affect the model’s performance. Additionally, many studies did not perform privacy attacks as a risk metric. This metric is essential for realistic dataset assessment and knowing whether it can stand against these attacks. This study will solve these limitations and challenges to create a more robust, privacy-preserving methodology. This study aims to address these gaps by using CTGAN with a tailored noise and dynamic adjustment that can maintain the data features and distribution without the risk of privacy attacks. Additionally, our study conducts extensive privacy attack tests to evaluate the robustness of the generated synthetic data. This research aims to advance the privacy–utility tradeoff in synthetic data generation and establish a more resilient framework for privacy-preserving synthetic data applications by focusing on these areas.

## 3. Methodology

**Figure 3:**
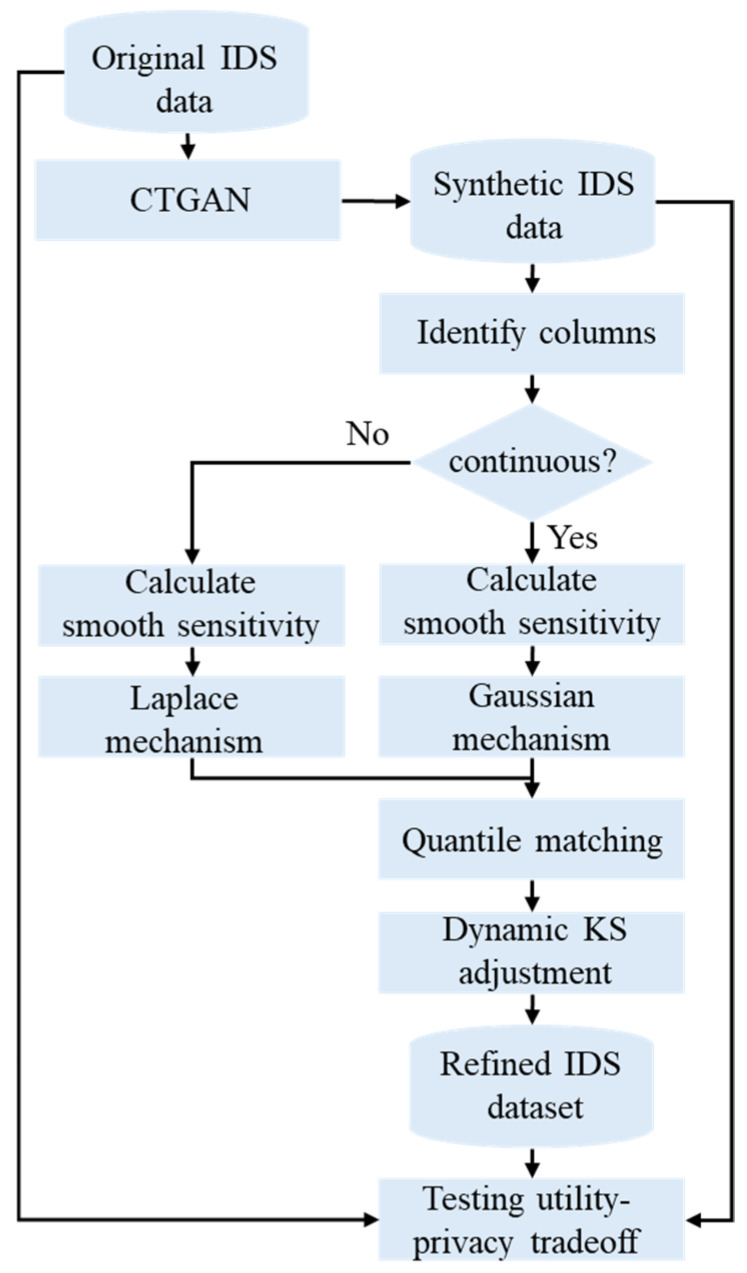
A flowchart of the overall utility-preserving DP method.

This section will describe the dataset and tools used to achieve the study’s goal. Additionally, it will define the utility and privacy metrics to ensure that the proposed method balances the utility–privacy tradeoff. Figure 3 shows the steps of performing the overall methodology; it starts with generating the dataset using CTGAN, then applying the DP method, and finally testing the utility–privacy tradeoff. The system configuration was 13th Gen Intel(R) Core(TM) i9-13900K (Intel Corporation, Santa Clara, CA, USA) and the CTGAN version was 1.17.1 [12].

### 3.1. Dataset

For the experiment, the MQTT-IoT-IDS2020 dataset was selected [23]. It was collected from a realistic IoT network that utilized the message queuing telemetry transport (MQTT) protocol, a lightweight messaging protocol connecting devices in machine-to-machine communication. This protocol is based on transmission control protocol/Internet protocol (TCP/IP). MQTT is widely used in IoT environments due to its low bandwidth and power consumption. It consists of a publish/subscribe model for sending and receiving and a broker that manages the message disruption. It can also support modern encrypted communications and authentication mechanisms such as client certificates.

Several studies have used the MQTT-IoT-IDS2020 dataset to achieve their goals for DL and ML tasks [24,25]. The dataset contains four attack types: aggressive scan (Scan A), user datagram protocol (UDP) scan (Scan sU), Sparta secure shell brute force (Sparta), and MQTT brute-force attack (MQTT BF). Table 2 shows the features used in the experiment with a sample of their values. After data balancing, the dataset had 15 columns, and the total number of the dataset was 238,678, with 119,339 for normal and attack samples. Table 3 demonstrates the number of each attack class and normal class.

### 3.2. CTGAN for Generating the Synthetic Dataset

The CTGAN generative model, used in this experiment and provided by the synthetic data vault (SDV) library [12], which was built on the PyTorch library [26], was designed to generate realistic synthetic tabular data [27]. The conditional generator can generate data based on specific conditions, ensuring that the data generated are similar to the original dataset. The CTGAN consists of two main components: the generator and the discriminator. The generator is a fully connected neural network that takes random noise z and a condition vector representing discrete columns aiming to produce an output $G\left(z\right)$ that closely represents the real data distribution. The generator tries to minimize the loss function, and it is defined as follows:

(1) $$L_{G}=-E_{z∼p_{z}}\left[\log D\left(G\left(z\right)\right)\right]$$

The second part is the discriminator; its goal is to distinguish between real tabular and synthetic data. The lower the correct distinction, the higher the possibility that the generator produces high-quality data that represent the real data x. The discriminator’s loss function $L_{D}$ is defined as follows:

(2) $$L_{D}=-E_{x∼P_{data}}\left[\log D\left(x\right)\right]-E_{z∼p_{z}}\left[\log\left(1-D\left(G\left(z\right)\right)\right)\right]$$

CTGAN samples the data conditionally by specifying the categorical and continuous columns instead of random data generation. The generator is trained on specific categorical columns, allowing for handling data imbalances by creating samples representing the under-represented classes. Privacy preservation can benefit from this framework because it can generate a dataset that might not be linked to the real entities from which the data came, with a high resemblance. Yet sometimes, it overfits or leaks secret information, which can make it vulnerable to privacy attacks like re-identification [28]. Therefore, the DP technique and state-of-the-art generators are needed to preserve privacy.

In this experiment, the CTGAN was trained with 100 epochs; the embedding passed the data to the generator with 128 dimensions. The generator and discriminator’s dimension was (256,256) with a learning rate of 2 × 10^−4^ and an Adam optimizer with a decay set to 1 × 10^−6^. The generator and discriminator’s settings were based on the original study that introduced the CTGAN model [27].

**Figure 4:**
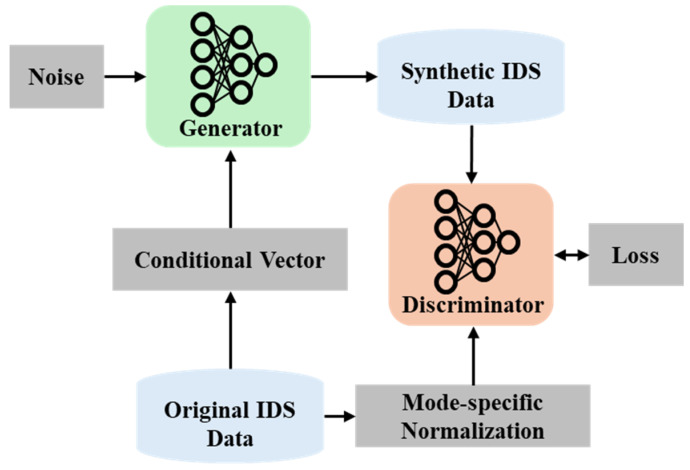
A flowchart of the CTGAN model.

As shown in Table 2, the generator’s output had two categorical features and 15 continuous features. The generator’s output will be used as a baseline for the synthetic data passed to the proposed DP method. UP-DP was not directly applied to the CTGAN model as it may lead to training instability. It was independently applied to the generated synthetic dataset. Figure 4 shows the flow of generating a synthetic dataset using a conditional generative model.

### 3.3. Utility-Preserved Differentially Private Synthetic Dataset

This section will explore advanced utility–privacy balancing. The proposed approach is to apply DP to the synthetic dataset while maintaining the utility tradeoff and the original dataset’s statistical properties. Instead of simply applying random noise that can affect the utility, two DP mechanisms were initially applied: Laplace for categorical data and Gaussian for continuous data to introduce noise into the dataset and ensure differential privacy. The Laplace mechanism was selected for the categorical data as it can inject noise within appropriate bounds and stepwise changes. While Gaussian noise was applied to the continuous data for its ability to be controlled with lower variance, small changes could significantly distort the results.

The noise was applied with smooth sensitivity to control the degree of the noise, which was determined by calculating the maximum absolute difference between the sorted adjacent values. The privacy budget parameter also scaled the Laplace noise, which was then added to the column values to generate noisy and differentially private data. After that, the noisy categorical and continuous data were combined again into one dataset.

After noise injection, quantile matching was performed, in which each column’s quantiles were aligned with the columns of the real data to maintain the overall distribution. This step ranked the values in the noisy and real datasets and then replaced each noisy value with its corresponding quantile from the real dataset. To prevent exact matches and overfitting, jittering was applied to the synthetic dataset, ensuring it maintained a level of privacy while approximating the real data distribution.

To further enhance the utility of the noisy data, dynamic adjustments were made using the Kolmogorov–Smirnov (KS) test, which measures the similarity between the distributions of real and synthetic datasets. First, the noisy and real data values were partitioned into bins, and the KS test was performed on these binned values. This step provided a statistic that quantified the dissimilarity distribution between datasets. Based on the output of the KS test statistics and the variance of the original data, the adjustment threshold was dynamically set. If the KS test statistics exceeded the threshold, the noisy data were modified by clipping values to be within the appropriate bin edges to better match the histogram of the original data. To avoid overfitting and privacy leakage, a slight noise was added probabilistically to maintain the DP while smoothing any sudden corrections during the process. Algorithm 1 describes the proposed utility-preserving DP methodology in detail.
**Algorithm 1.** The steps for performing UP-DP to the synthetic dataset.**Input:****Synthetic data** containing discrete and continuous columns.**Output:**Differentially private and utility-preserved **synthetic data**1:Apply Differential Privacy2:
Partition the columns into **discrete** and **continuous**3:
Apply smooth sensitivity-based Laplace noise for discrete columns4:

For each **column** in **discrete**5:


Compute **smooth_sensitivity** as the maximum absolute difference6:

Apply Laplace noise7:
Apply Gaussian noise for continuous columns8:

For each **column** in **continuous**9:


Calculate the range of the column values10:


Compute column sensitivity as a fraction **sensitivity_factor** of this range12:


Apply Gaussian noise13:
Combine noisy **discrete** and **continuous** data14:Apply Quantile Matching15:
Match **noisy data** quantiles16:
Add slight jitter to avoid overfitting17:Dynamic KS Adjustment18:
Compute binned distributions19:
Dynamically adjust **noisy data** based on KS statistic20:

Clip values of the **noisy data** within the bin edges21:Return the **utility-preserving DP synthetic data**

To test this step, the utility–privacy tradeoff metrics were measured against the synthetic data before and after the utility-preserved DP. It was also compared to a synthetic dataset that applied several well-known standard DP methods as a baseline without focusing on the utility aspects: Laplace, exponential, and Piecewise. The baseline DP methods utilized a uniform noise level across data features. The Laplace mechanism adds noise obtained from the Laplace distribution to numeric data, scaling it to balance privacy and utility [29]. The exponential mechanism applies DP by selecting outputs probabilistically based on utility scores [30]. The Piecewise mechanism divides data into segments, adding noise within each segment to preserve natural data patterns. It offers a more targeted privacy approach for data with varying sensitivity, enhancing both privacy and utility [31]. As raw noise can achieve the lowest privacy risk, the results of these methods were used as a threshold to identify whether our proposed method can achieve low privacy risks.

### 3.4. Privacy and Utility Metrics

In order to test the privacy risk resilience of the proposed method, we considered testing them against privacy attacks and used statistical and ML performance metrics for the utility test. This part categorizes the performance measures into utility and privacy metrics. Multiple metrics were selected to perform both measures; each had its goal and focus on determining the utility and privacy performance.

#### 3.4.1. Utility Metrics

The utility metrics aim to determine whether the synthetic dataset resembles the original dataset. It is essential as the downstream tasks need high-quality data to be deployed effectively. In the context of ML-based IDSs, the attack attributes should not be overshadowed by the overprocessing of the DP technique, as it can lead to high error rates [32]. The Kolmogorov–Smirnov (KS) test is a widely used method of measuring distribution similarities. It compares two cumulative distribution functions (CDFs) by measuring the largest vertical distance between them among all instances, and it is calculated as follows:

(3) $$D=\underset{x}{\sup}\left|F_{1}\left(x\right)-F_{2}\left(x\right)\right|$$

where *D* is the KS statistic, representing the maximum difference between the CDFs’ suplex overall values x. The $F_{1}\left(x\right)$ and $F_{2}\left(x\right)$ are the empirical CDFs of the first and second samples. The KS test version used was the KS complement, as it has been used in tabular GAN-related research [33,34,35]. The KS complement was calculated as 1−D, which made the KS test a similarity measure instead of a difference measure ranging from 1.0 to 0.0.

While the KS test measures the largest deviation between two distributions, the Jensen–Shannon divergence (JSD) evaluates the overall similarity between the probability distributions of the original and synthetic data. It is a symmetric measure of similarity between two probability distributions, *P* and *Q*, based on the Kullback–Leibler divergence (KLD). The formula of the JSD is as follows:

(4) $$\mathrm{JSD}\left(P∥Q\right)=\frac{1}{2}D_{\mathrm{KL}}\left(P∥M\right)+\frac{1}{2}D_{\mathrm{KL}}\left(Q∥M\right)$$

where $M=\frac{1}{2}\left(P+Q\right)$ is the average of the two distributions *P* and *Q*.

Another metric to ensure the data’s utility is the Wasserstein distance, also known as Earth Mover’s distance (EMD). It measures the distance d between two probability distributions *P* and *Q* over a given metric space, the Wasserstein-1 distance in this case, and it is calculated as follows:

(5) $$W_{1}\left(P,Q\right)=\underset{\gamma \in \Pi \left(P,Q\right)}{\inf}E_{\left(x,y\right)∼\gamma }\left[d\left(x,y\right)\right]$$

where $\Pi \left(P,Q\right)$ is a set that contains all possible joint distributions, also known as couplings $\gamma \left(x,y\right)$.

An additional utility metric to consider was ML performance; it assesses how well the synthetic data can be used in the downstream tasks. The main goal of creating the MQTT-IoT-IDS2020 dataset was to use it in intrusion detection tasks, such as ML-based IDS. This experiment used the MLP model provided by the scikit-learn library [36], to classify the attack and normal network packets. Researchers have shown that MLP classifiers can accurately classify the network packets on the IoT networks dataset for IDSs [24,37,38]. Hence, the proposed model should also show high accuracy to ensure utility preservation. The MLP is configured with a hidden layer size of 50, a regularization with 0.01 to avoid overfitting, a 0.001 learning rate, and 100 epochs. The dataset was split into 80% synthetic for training and 20% of the original data for testing. The datasets were preprocessed using standardization before being passed to the classifier to ensure fair feature treatment, as the MLP classifier can be biased toward features with a more extensive range.

The synthetic data were measured using the train-on-synthetic, test-on-real (TSTR) approach to ensure that the synthetic data could replace the original data. The ML performance metrics were accuracy and the F1 score, based on the confusion matrices of classification models. The accuracy and F1 score are calculated as follows:

(6) $$\mathrm{Accuracy}=\frac{True\;Positives+True\;Negatives}{Total\;Number\;of\;Samples}$$

(7) $$F1=\frac{2\times True\;Positives}{2\times True\;Positives+False\;Positives+False\;Negatives}$$

#### 3.4.2. Privacy Metrics

The privacy metrics aim to determine whether the synthetic datasets are robust against privacy attacks. This metric measures the probability of successful attacks. Several attacks might cause privacy risks, and performing the attacks is needed to test these risks. This experiment covered different types of attacks, including singling out, linkability, and inference. These attacks were performed using the methods provided by the Anonymeter library [39], and they are key privacy risk measures according to the European General Data Protection Regulation (GDPR) [40].

Singling out is a privacy risk that tries to identify a unique entity in the dataset even when their related information does not exist; by combining different data points, when the attacker looks at them together, it points to a specific entity [41]. In the experimental setting, the confidence level was set to 0.95 based on the source code in [34], which is the probability that a confidence interval contains the true value of a measured singling-out risk.

The linkability risk occurs when the attacker tries to associate several attributes’ values with the same entity without knowing its identity [42]. The attribute inference risk is when an attacker tries to infer an entity’s private or sensitive, not explicit attribute from known attributes [43]. This privacy risk shows how analyzing non-sensitive data can reveal a privacy breach. Attribute inference and linkability attacks are performed using quasi-identifiers, which are attributes accessible to an attacker. Although not directly tied to specific entities, these attributes can be combined to link records. In this experiment, the selected known attributes were source and destination ports, which, while generally stable within a single communication session, vary across sessions and users; protocol, as each protocol exhibits unique traffic patterns; and the mean and standard deviation of packet length, which capture the general network flow and reflect the typical data size for specific behaviors. These attributes were chosen to evaluate their stability and potential sensitivity in the context of linkability and attribute inference risks. These attacks were performed on synthetic data before and after DP and compared to the UP-DP method.

## 4. Results

In the utility–privacy tradeoff, we need to ensure that the differentially private dataset resembles the original dataset while maintaining low privacy risks. This section will measure the proposed method’s performance in terms of utility preservation using statistical and ML metrics. Then, privacy risks will be performed to test how the proposed method can synthesize a dataset that can resist privacy attacks with a low success rate. The synthetic data without DP was first tested as a baseline, as the DP technique did not affect its utility.

### 4.1. Utility Preserving Results

The KS test, JSD, and Wasserstein distance were performed to measure the utility preserving performance and compare the original and synthetic data. The results in Table 4 show that the synthetic data without DP and with UP-DP are highly similar in terms of the maximum differences between the distributions (0.79 and 0.80, respectively), a low divergence between the probability distributions with 0.32, and a low cost of transforming one distribution into another with 0.17, unlike the synthetic data without UP-DP that showed 0.68 in the KS test, which demonstrates that the DP affected its similarity. These results show that the privacy measures did not affect the CTGAN performance in generating a synthetic dataset that highly resembles the real dataset.

**Figure 5:**
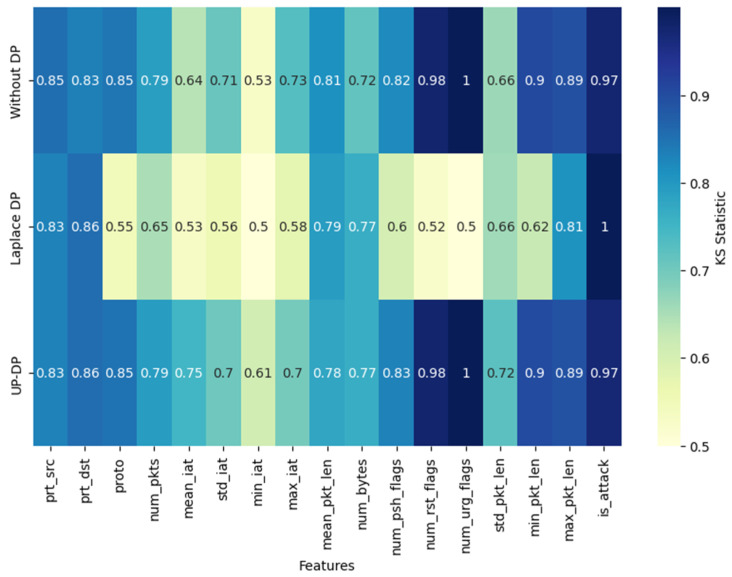
A heatmap of the KS test scores per feature for different synthetic data.

Figure 5 is a heatmap that compares the KS test score of each feature for the three settings: synthetic data without DP, with Laplace DP, and with UP-DP. It demonstrates that the KS clipping step in UP-DP can increase the KS test score of features such as std_pkt_len, min_iat, and num_bytes. However, a few features showed lower KS scores, which can be due to the last step of applying jittering to avoid overfitting and matching with the original data.

Regarding the performance of the ML model, the MLP in Table 5 showed a high accuracy with 0.9943 using the synthetic data without the DP, which indicates that the CTGAN can generate a high-quality dataset that can be used as a replacement for the original data. On the other hand, performing DP significantly affected the dataset’s quality with 0.8353 accuracy. Applying the utility-preserving technique to the DP significantly helped improve the accuracy of the MLP model to 0.9253. While DP alone can degrade model performance, integrating utility-preserving techniques is essential to maintain the usefulness of synthetic data in sensitive scenarios, enabling a more optimal tradeoff between privacy and data utility. These results indicate a linear trend in utility damage, where adding the DP mechanism alone causes a notable drop in performance metrics compared to the synthetic data generated without privacy constraints. It reflects a direct relationship between added privacy constraints and reduced model performance. The improved performance with UP-DP suggests that the utility-preserving technique effectively mitigates some of the accuracy loss by retaining more informative patterns within the synthetic data about the features of the attack and normal packets, even under privacy constraints. This linkage between utility-preserving modifications and improved model performance highlights the effectiveness of UP-DP in balancing privacy and utility, as reflected in its higher accuracy and F1 scores compared to DP mechanisms alone.

Compared to the related work by [20], the quality of the synthetic data generated by CTGAN was not affected by the UP-DP method. Furthermore, some features were improved through KS test clipping. The high accuracy of the synthetic dataset, measured using machine learning performance metrics, along with the high similarity in statistical metrics, indicates that the isolated CTGAN model can generate high-quality data without being influenced by directly linking the DP method to it, as applied by [19].

**Figure 6:**
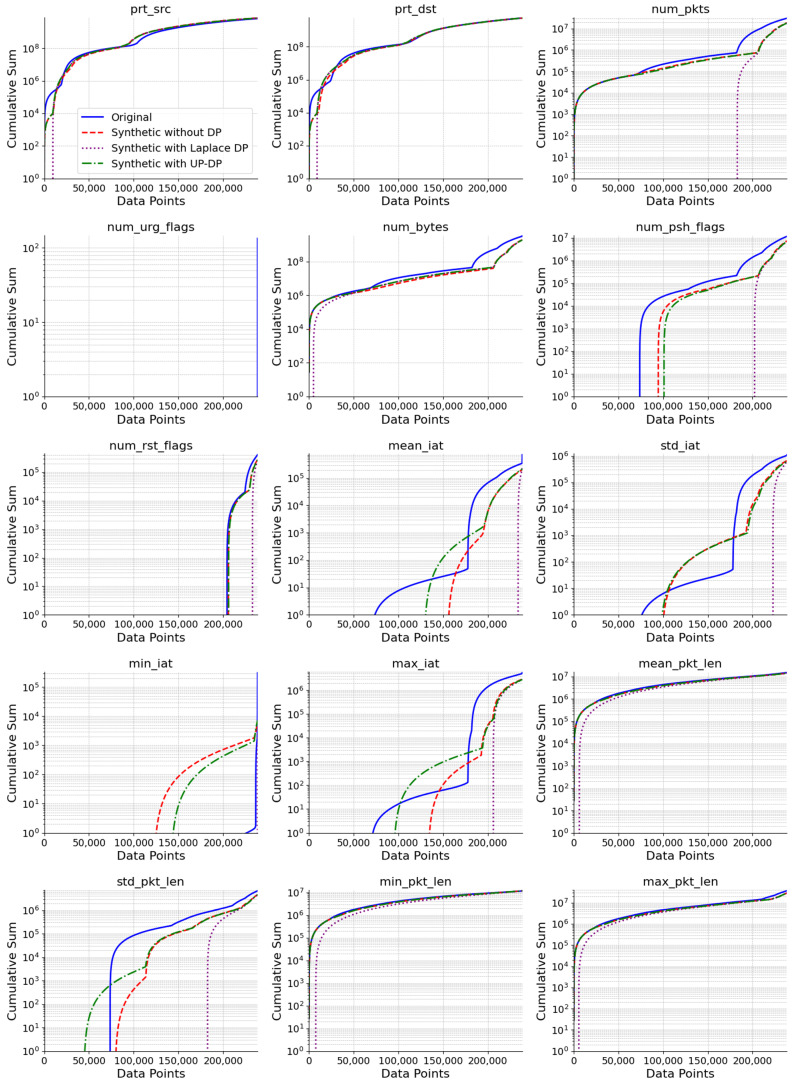
The log-scaled cumulative sum of each numerical feature compares the fit of each synthetic dataset with the original data.

To visualize the results, the cumulative sums illustrate how well the proposed synthetic dataset with UP-DP fits with the original dataset and prove that it maintains the attack and normal packet’s features. The cumulative sum is a sequence of sums of given data; it is the sum of all the index’s elements. Researchers use it as a testing tool to obtain a visual understanding of the synthetic datasets and how they are affected by the experimental settings [33,44,45]. Figure 6 shows a detailed comparison between the original data and the three synthetic data types, without DP, with Laplace DP, and UP-DP. The Laplace DP was selected for comparison as it showed the highest accuracy across the common DP mechanisms. The cumulative sums of each feature clearly demonstrate that the synthetic data with the UP-DP method was closer to the original dataset and maintains its characteristics, unlike the synthetic data with Laplace DP, which showed a significant difference in shape from the original data in most features. The synthetic data without any DP was the closest fit to the original data compared to the rest of the synthetic data.

### 4.2. Privacy Risks Results

Privacy risk metrics assess the effectiveness of anonymization by evaluating vulnerabilities to attacks like singling out, linkability, and attribute inference. The objective is to minimize privacy risk as much as possible. The DP mechanisms, Laplace, exponential, and Piecewise, were compared to the proposed method. While a robust DP method can greatly reduce privacy risks, it also impacts utility. Therefore, this experiment aims to develop a method that preserves utility while achieving privacy risk results similar to DP alone.

Table 6 compares the privacy risks associated with each synthetic data method, highlighting the effectiveness of the DP and UP-DP approaches. In the case of singling out, the DP method nearly eliminated the risk, reducing it from 0.99 to 0.0009. Similarly, the UP-DP method significantly reduced the singling-out risk to 0.0069 while maintaining data utility. Linkability also decreased considerably, with the Laplace mechanism method reducing the risk from 0.70 to 0.003,and the UP-DP method further reducing it to 0.001. For attribute inference, the Laplace mechanism significantly reduced the risk from 0.98 to 0.57, and UP-DP achieved a further reduction to 0.35. While the exponential mechanism showed the lowest privacy risks in linkability and inference attacks, it showed the lowest performance in the ML performance metric.

Overall, UP-DP reduced privacy risks across all attack types by over 90% compared to the baseline without DP, closely approaching the performance of the DP methods alone. This indicates that UP-DP provides a promising solution for minimizing privacy risks close to the common DP methods without significantly compromising data utility.

## 5. Discussion

The results obtained from the proposed CTGAN-based privacy-preserving synthetic data generation approach underscore the effectiveness of the UP-DP methodology. This section will evaluate the significance of these findings and the implications for using synthetic datasets in IoT-based IDS. Additionally, it will address the limitations and potential future improvements.

One of the main challenges when using synthetic data and DP is achieving a balance between privacy and utility, especially when dealing with third parties or publishing network datasets for researchers to contribute to the ML-based IDS field. The experimental results indicate that utilizing UP-DP improved the utility of the differentially private dataset while maintaining low privacy risks. The KS test of 0.80 suggests that the dataset remains highly representative of the original distribution despite introducing noise for privacy purposes. The metrics such as JSD and Wasserstein distance further support this outcome, demonstrating minimal degradation in data quality. Moreover, the TSTR test on the synthetic datasets, the UP-DP dataset, was much closer to the non-private data than using standard DP.

While studies such as [19,20] focused on incorporating DP into GAN models, they often suffered from issues like training instability or a significant loss of data utility due to random noise injection. In contrast, quantile matching and KS adjustment allowed better preservation of the original dataset’s statistical properties. Table 7 compares different methods for generating IDS datasets, focusing on their privacy aspects and performance measured by the KS test. CTGAN and Copula GAN are used to generate generic IDS datasets, but they lack a focus on privacy, showing KS test scores between 0.77 and 0.95. In contrast, the proposed method, UP-DP, uses CTGAN to generate IoT-specific datasets and incorporates privacy-preserving techniques, addressing privacy risks such as singling out, attribute inference, and linkability. UP-DP achieved a competitive KS test score of 0.80 while enhancing privacy protection, distinguishing it from other approaches.

Despite these improvements, the study has certain limitations. While UP-DP improved utility compared to standard DP, there was still a performance drop compared to the synthetic data generated without any privacy mechanisms. This underscores the fundamental challenge of preserving utility when applying DP, where even advanced methods like UP-DP cannot entirely balance the tradeoff between utility and privacy. Furthermore, although the privacy risk metrics showed substantial reductions, there remains a need for further refinement, particularly in ensuring robustness against more sophisticated privacy attacks.

These findings imply that it is possible to significantly mitigate the privacy risks associated with sharing synthetic data from IoT sensor networks without completely sacrificing utility. This provides more widespread use of synthetic data in privacy-sensitive applications like IDS, where sharing real datasets might cause privacy risks. Additionally, the success of UP-DP in improving the utility–privacy tradeoff suggests that future research should focus on refining these techniques further to close the gap between private and non-private synthetic data.

Future work should explore more advanced DP mechanisms, using adaptive privacy budgets or personalized privacy settings based on the network packets’ features to tailor noise injection based on their sensitivity. Additionally, extending the evaluation to include more diverse IoT datasets and real-world privacy attacks would provide a more comprehensive evaluation of the proposed method’s robustness.

## 6. Conclusions

Extracting private information from network datasets is a serious privacy concern. Attackers can extract user behavior, like daily routines and work habits, from traffic patterns. Researchers and organizations must take precautions, such as anonymization and DP, and ensure compliance with regulations such as GDPR to protect private information. However, protecting private information comes with the challenge of the utility–privacy tradeoff, which needs to be studied and addressed to close the gap between them.

This study demonstrates the effectiveness of a CTGAN-based method for generating privacy-preserving synthetic data, balancing the tradeoff between utility and privacy in IoT sensor network intrusion detection. By integrating differential privacy with advanced noise control mechanisms, such as dynamic KS adjustment and quantile matching, the statistical properties of the original dataset were preserved while reducing privacy risks across various attack types. The proposed method maintained a high utility–privacy balance, outperforming the traditional DP technique, and is thus a promising solution for secure data sharing in IoT environments. Future research should focus on enhancing privacy mechanisms to mitigate advanced attack vectors and expanding evaluations to a broader range of datasets and real-world scenarios.

## Figures and Tables

**Table 1:** A systematic review of the related works and their limitations.

Study	Method	Key Features	Limitations
Venugopal et al. (2022) [10]	pGAN	Batch normalization in the generator and dropout in the discriminator to improve convergence	The data generated were not tested against privacy attacks
Rajabi et al. (2022) [15]	TabFairGAN	Improving the fairness of GAN models	Did not measure the privacy metrics
Fang et al. (2022) [18]	DP-CTGAN	Random perturbation added and tracking the privacy loss	The privacy measures affected the performance of the CTGAN model
Kroes et al. (2023) [22]	CBSDG	Removing intra-cluster relationships rather than adding noise	The method was not tested against privacy attacks
Nik et al. (2023) [16]	GAN methods	Compared several tabular GANs	The privacy attacks were not measured and differential privacy was not applied to the GANs
Hindistan and Yetkin (2023) [20]	GAN and DP	Adding Laplace distribution noise to the sensitive features	Applying random noise reduces the utility of the dataset
Sun et al. (2023) [19]	DP-CGANS	Injected Gaussian noise into the gradient updates during training	Adding noise directly may lead to training instability
Almeida and Bacao (2024) [21]	UMAP-SMOTENC	Combining dimensionality reduction with oversampling	Did not consider the use of privacy risk metrics
Sakib and Ghosh (2024) [17]	GAN methods	Compared several tabular GANs	It does not explore advanced utility–privacy balancing

**Table 2:** Features’ definitions and data types.

Feature	Data Type	Definition	Sample Value
proto	Categorical	Transport layer protocol	TCP
num_pkts	Integer	Number of packets	330
mean_iat	Decimal	The mean of inter-arrival time	3.03
std_iat	Decimal	The standard deviation of the inter-arrival time	26.85
min_iat	Decimal	The minimum inter-arrival time	4.05
max_iat	Decimal	The maximum inter-arrival time	89.10
num_bytes	Integer	Number of bytes	272
num_psh_flags	Integer	Number of push flags	75
num_rst_flags	Integer	Number of reset flags	1
num_urg_flags	Integer	Number of urgent flags	0
mean_pkt_len	Decimal	The average of the packet length	69.57
std_pkt_len	Decimal	The standard deviation of the packet length	3.2
min_pkt_len	Decimal	The minimum packet length	52.0
max_pkt_len	Decimal	The maximum packet length	60.0
is_attack	Categorical	1 for attack and 0 for normal	1

**Table 3:** The number of instances per class.

Class	Number
Scan_A	46,473
MQTT_bruteforce	31,311
Sparta	118,374
Scan_sU	42,520
Normal	119,339
Total	238,678

**Table 4:** The methods’ performance on statistical utility metrics.

Synthetic Data Method	Utility Metrics		
	KS Test ↑	JSD ↓	Wasserstein Distance ↓
Without DP	0.79	0.32	0.08
Laplace mechanism	0.68	0.51	0.17
Exponential mechanism	0.38	0.34	0.09
Piecewise mechanism	0.38	0.60	0.59
UP-DP	**0.80**	**0.32**	**0.08**

**↓** = lower is better, **↑** = higher is better, bold indicates the best performance.

**Table 5:** The performance of the methods on the MLP classifier, based on accuracy and the F1 score.

Synthetic Data Method	ML Performance Metrics	
	Accuracy	F1 Score
Without DP	0.9943	0.9943
Laplace mechanism	0.8353	0.8180
Exponential mechanism	0.4726	0.6418
Piecewise mechanism	0.4781	0.6412
UP-DP	0.9253	0.9246

**Table 6:** Comparison of privacy risks in synthetic data methods.

Synthetic Data Method	Privacy Risks ↓		
	Singling Out	Linkability	Inference
Without DP	0.99	0.70	0.98
Laplace mechanism	0.0009	0.003	0.57
Exponential mechanism	0.0005	0.0009	0.19
Piecewise mechanism	0.0003	0.0014	0.22
UP-DP	0.0069	0.001	0.35

**↓** = lower is better.

**Table 7:** Comparison of IDS dataset generation methods.

Method	Type of IDS Dataset	Generator Type	Privacy Attack Tests	KS Test ↑
[4] (2022)	Generic	CTGAN	No	0.77 to 0.82
[46] (2022)	Generic	CTGAN	No	0.85
[47] (2023)	Generic	Copula GAN	No	0.85
[33] (2023)	IoT	CTGAN	ML adversarial attack	0.72
[35] (2024)	Generic	Copula GAN	No	0.95
Proposed Method	IoT	CTGAN	Singling out attribute inference, Linkability	0.80

**↑** = higher is better.

## Data Availability

The network dataset MQTT-IoT-IDS2020 is publicly available and can be downloaded from: https://ieee-dataport.org/open-access/mqtt-iot-ids2020-mqtt-internet-things-intrusion-detection-dataset (accessed on 1 October 2024).
